# Bifunctionalized Polyethyleneimine-Based Sponge for Adsorption of Ibuprofen from Aqueous Solution

**DOI:** 10.3390/polym17233221

**Published:** 2025-12-03

**Authors:** Xiaoyi Gou, Zia Ahmad, Zaijin You, Zhou Ren

**Affiliations:** 1 College of Transportation Engineering, Dalian Maritime University, Dalian 116026, China; 2 Centre for Ports and Maritime Safety, Dalian Maritime University, Dalian 116026, China; 3 Dalian National Laboratory for Clean Energy, Dalian Institute of Chemical Physics, Chinese Academy of Sciences, 457 Zhongshan Road, Dalian 116023, China

**Keywords:** anion exchange interaction, anionic NSAIDs, polyethyleneimine

## Abstract

A quaternized and phenyl-functionalized hyperbranched PEI-based sponge (S_HPEI-QP_) was successfully prepared, and its adsorption performance was investigated to evaluate its potential for removing the anionic non-steroidal anti-inflammatory drug (ibuprofen (IBU)). We reported that the synthesis of polyethyleneimine-based sponges was achieved through cryo-polymerization using 1,4-butanediol diglycidyl ether (BDDE) as the crosslinking agent. Subsequent functionalization with resorcinol diglycidyl ether (RDGE) and trimethylamine introduced quaternary ammonium cations, imparting strong basicity and hydrophilicity, as well as phenyl groups, conferring hydrophobic characteristics, respectively. The aforementioned sponge material, S_HPE-QPI_, primarily facilitates the efficient adsorption of IBU in aqueous solutions through the anion exchange properties of quaternary ammonium groups and the π-π interactions associated with oxygen-activated benzene rings. Various characterizations, such as scanning electron microscopy (SEM), Fourier transform infrared spectroscopy (FT-IR), X-ray photoelectron spectroscopy (XPS), and specific surface area determination method (BET), confirmed the successful synthesis of the bifunctionalized S_HPEI-QP_ adsorbent. This adsorbent features a porous structure (specific surface area of 77.2 m^2^ g^−1^ and pore size distribution of 25–100 nm) and an isoelectric point (pH_pzc_) of 9.38. The adsorption kinetics of the adsorbent for IBU were extremely rapid and conformed to a pseudo-second-order kinetic model, and the adsorption isotherm aligned with the Langmuir isotherm model. Noteworthily, S_HPEI-QP_ demonstrated an exceptionally high adsorption capacity for IBU, achieving a maximum uptake of 905.73 mg g^−1^ at pH 7.0, which surpassed that of most of the previous reported adsorbents. Moreover, the sponge material can be chemically regenerated. After eight cycles of use, the adsorption efficiency decreased by only 4%. These findings suggest that the synthesized dendritic anion exchange adsorbent represents a promising candidate for the removal of IBU from contaminated water sources.

## 1. Introduction

Pharmaceutically active compounds (PhACs), identified as a category of emerging contaminants, have attracted considerable attention from both the scientific community and the general public due to their persistent release and potential toxicological risks to human health and wildlife. As a result, these substances have become a core research focus within the fields of environmental science and engineering disciplines [1,2]. Several factors collectively drive the widespread contamination of aquatic environments by pharmaceuticals [3]: the pervasive and frequent utilization of pharmaceuticals, excretion by humans and animals, improper disposal of unused medications, and inherent limitations in wastewater treatment technologies. This issue has elicited significant international concern, as the continuous and enduring discharge of pharmaceuticals into water bodies gives rise to persistent pollution within aquatic ecosystems [4].

Non-steroidal anti-inflammatory drugs (NSAIDs) are regarded as one of the most extensively used pharmaceutical classes worldwide, encompassing compounds such as aspirin, acetaminophen, ibuprofen, diclofenac, and naproxen. Given their widespread production and consumption, NSAIDs are notably prevalent as residual contaminants in aquatic environments and have been consistently detected in various water bodies. For example, various NSAIDs, including acetaminophen, diclofenac, and aspirin, have been reported in the effluents from wastewater treatment plants in countries such as Italy [5], Thailand [6], and the United States [7]. In Japan, surface water concentrations of NSAIDs have been recorded with maximum and average values of 1889 ng L^−1^ and 244 ng L^−1^, respectively [8]. Similarly, ibuprofen concentrations in surface waters in the United States have reached levels up to 36,788 ng L^−1^ [9], while acetaminophen, diclofenac, naproxen, ketoprofen, and indomethacin have been detected at nearly 1 μg L^−1^ [10]. NSAIDs exhibit bioaccumulative properties in living organisms, and despite typically occurring at trace concentrations in the environment, their persistent presence poses ecological risks [11,12]. To be specific, empirical evidence indicates that NSAIDs in aquatic environments induce chronic toxicity in biota [13,14], diminish planktonic species diversity [15], and extremely disrupt the community structure of aquatic organisms and microorganisms [16]. In order to reduce their harmful effects, the development of effective strategies is in high demand for the removal of NSAIDs from the aquatic environment.

Physicochemical processes, biochemical treatments, and their integrated systems stand as the primary approaches for removing the emerging contaminants (such as NSAIDs) in wastewater [17,18,19]. However, these treatment strategies present several limitations, including prolonged degradation times, the requirement for secondary chemical additives, and the potential generation of secondary pollution, all of which result in suboptimal treatment efficacy. For instance, ibuprofen predominantly exists in aqueous environments as negatively charged ions, while the surfaces of microorganisms and algae are also similarly negatively charged [20]. This electrostatic repulsion thereby impedes the adsorption and metabolic processing of ibuprofen by these biological agents. Moreover, the intrinsic biotoxicity of ibuprofen narrows the applicability of biological treatment methods to NSAID-contaminated wastewaters within specific concentration ranges. Research has demonstrated that although conventional biological treatments can achieve ibuprofen degradation, certain microorganisms may produce metabolites such as hydroxy-ibuprofen and carboxy-ibuprofen, which exhibit greater biotoxicity than the parent compound.

Adsorption is extensively utilized in water treatment due to its simplicity, cost-efficiency, lack of by-product formation, and superior effectiveness relative to biodegradation and advanced oxidation methods. The efficacy of adsorption is predominantly dependent on the selection of adsorbent materials, which constitutes a critical domain of ongoing research and investigation [12]. The selection of a suitable adsorbent is critical to attaining optimal adsorption efficiency. A wide variety of adsorbents have been employed in adsorption processes, including carbon-based adsorbents, polymeric resins, metals and their oxides, as well as molecularly imprinted polymers [21,22,23]. For instance, Wei et al. developed a novel granular composite adsorbent by combining carbon nanotubes (CNTs) with the surfactant Brij35 and alumina (Al_2_O_3_), followed by calcination to remove the surfactant, resulting in porous particles in virtue of excellent adsorption and regeneration capabilities. This composite was effectively applied for the adsorption of diclofenac sodium and carbamazepine from aqueous solutions [24]. Similarly, Luo et al. fabricated a three-dimensional metal–organic framework functionalized with carboxyl groups as an adsorbent, which efficiently removed pharmaceuticals such as diclofenac sodium and chlorpromazine hydrochloride from water [25]. Furthermore, Liu et al. synthesized an environmentally friendly adsorbent based on a zeolitic imidazolate framework-67 molecularly imprinted polymer composite (ZIF-67-MIP), capable of simultaneously adsorbing three non-steroidal anti-inflammatory drugs-diclofenac sodium, flunixin meglumine, and nimesulide [26]. However, the significant improvement of adsorption efficiency is still largely restricted by the lack of strong binding sites for target pollutants.

Hyperbranched polyethyleneimine (PEI) has been widely used as an excellent adsorption matrix for the treatment of heavy metals and organic pollutants in wastewater [27]. Featuring abundant primary and secondary amine groups in its backbone, in a typical hyperbranched polymer, the monolithic adsorbent derived from PEI integrates its superior adsorption performance with simple separation from the solution. Sponge materials possess distinctive characteristics, such as high wettability, a large number of terminal groups, and internal molecular functional groups, which facilitate the penetration of wastewater into the adsorbent and ensure thorough contact, thereby enabling efficient and rapid adsorption of pollutants [28]. Soumyajyoti et al. developed macroporous polymer sponges through ice templating of aqueous PEI solutions, followed by crosslinking under frozen conditions [29]. Zia et al. proposed the synthesis of bifunctionalized PEI-based sponges, which, through bifunctional modification, provided both quaternary ammonium cations and phenyl functional groups for the highly selective removal of anionic drugs (diclofenac), dyes (methyl orange), and Cr(VI) [30]. Despite the reported modification of functionalized sites, there are still problems, such as the long time required to reach adsorption equilibrium and low adsorption capacity. This study aims to improve the preparation of a hyperbranched functionalized sponge that can quickly reach adsorption equilibrium and has a higher adsorption capacity. Hyperbranched functionalized sponge is an effective building block in adsorption chemistry by virtue of a high density of functionalities, as well as large and accessible pore structures.

In this study, a hyperbranched functionalized sponge adsorbent was synthesized via crosslinking and grafting techniques to achieve efficient removal of ibuprofen (IBU) from aqueous solutions. This study is the first to use cross-linked PEI sponge as a substrate and further graft PEI on the sponge to provide more modifiable sites. Finally, the hyperbranched sponge was bifunctionalized. The developed adsorbent exhibited notable properties, including high adsorption capacity, excellent reusability, and cost-effectiveness. Specifically, to introduce a higher density of amino functional groups, high-molecular-weight polyethyleneimine (PEI) was first grafted onto the surface of the sponge-like matrix. Subsequently, additional functional groups were incorporated into the polymer sponge through reactions with resorcinol diglycidyl ether and methylamine. To evaluate the adsorption behavior of ibuprofen (IBU) on the sponge and clarify the underlying mechanism, systematic studies were conducted, covering adsorption kinetics, thermodynamics, selectivity, and reusability. Compared with conventional adsorbents, the hyperbranched functionalized sponge offers multiple distinct advantages: first, its reticular sponge-like structure facilitates the penetration of wastewater, ensuring comprehensive contact and efficient adsorption; second, the three-dimensional architecture of hyperbranched polymers imparts unique characteristics such as high solubility, abundant terminal groups, and internal functional moieties, rendering them promising candidates for adsorption materials, drug delivery systems, and other functional carriers; third, the abundant functional groups in bifunctionalized polymer material facilitate effective removal of IBU via anion exchange interactions. Consequently, the synthesized bifunctionalized adsorbent demonstrates considerable potential for the adsorption and elimination of IBU in aquatic environments.

## 2. Experimental

### 2.1. Chemicals and Materials

Two HPEI (average Mw~1000 Da and~70,000 Da) were supplied by Sigma-Aldrich (Milwaukee, WI, USA). 1,4-butanediol diglycidyl ether (BDDE, 98%) was obtained from Alfa Aesar (Tianjin, China). The ibuprofen (IBU, >98%) was obtained from Tokyo Chemical Industry (TCI, Tokyo, Japan). Sodium hydroxide (NaOH), nitric acid (HNO_3_), and methanol were supplied by Beijing Chemical Works (Beijing, China). Resorcinol diglycidyl ether (RDGE, 94%) was from J&K Scientific (Beijing, China), and trimethylamine (30% aqueous solution), hydrochloric acid, and toluene were from Kermel Chemical Reagent Co. (Tianjin, China). The water used in all experiments was deionized water from a Milli-Q purification system (Billerica, MA, USA). HPLC-grade methanol was supplied by Fisher Scientific (Fair Lawn, NJ, USA). All the chemicals were used as received without further purification. The physicochemical properties and molecular structure of IBU are summarized in Table 1.

### 2.2. Preparation of Quaternized and Phenyl-Functionalized Hyperbranched PEI Sponge (S_HPEI-QP_)

**Figure 1:**
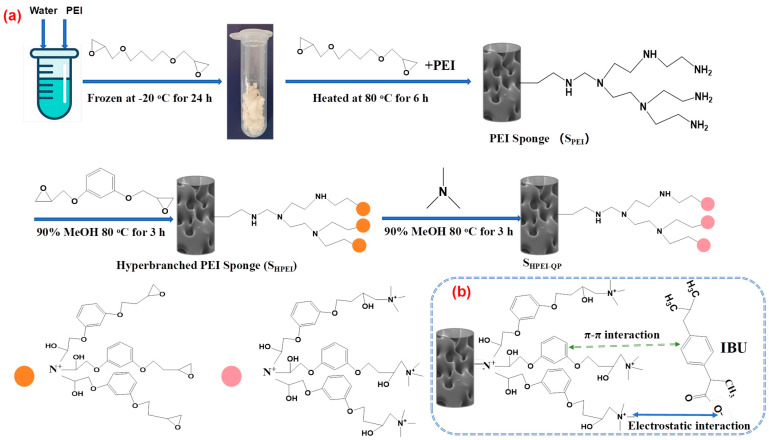
(**a**) Schematic illustration for the preparation of S_HPEI-QP_ and (**b**) the possible interaction mechanisms between DIC and the adsorbent.

A cryo-polymerization method was employed to synthesize polyethyleneimine (PEI)-based sponges via epoxy-amine reactions (Figure 1). In a representative procedure, 1.4 g of water and 1 g of a PEI (average Mw~1000 Da) water solution (containing 2.38 mmol of CH_2_CH_2_N units) were mixed and vortexed in an Eppendorf tube. Subsequently, 0.3 g of 1,4-butanediol diglycidyl ether (BDDE) was added as a cross-linking agent to yield a homogeneous mixture. The precursor solution was immediately subjected to cryo-polymerization by storage at −20 °C for 24 h. The resulting PEI sponge was obtained by thawing at ambient temperature, followed by extensive washing with water five times. The PEI-based sponge(S_PEI_) was functionalized by, respectively, immersion in a 20 mL 5%, 10%, 15% PEI (average Mw~70,000 Da) methanol–water (*v*:*v* = 50:50) solution and heated in water bath to 80 °C for 4 h, followed by repeated washing with water (five times) and drying under vacuum at 50 °C for 12 h, yielding the modified sponge designated as S_HPEI_ (hyperbranched PEI Sponge). Subsequently, the S_HPEI_ obtained in the previous step was reacted with 5 mL of resorcinol diglycidyl ether (RDGE) in a 20 mL methanol–water (*v*:*v* = 50:50) solution and heated in a water bath to 80 °C for 3 h, followed by repeated washing with water (five times). The epoxy-terminated S_HPEI_ was then treated with trimethylamine in 20 mL methanol–water mixtures (*v*:*v* = 50:50) at 80 °C for an additional 3 h. Upon completion of the reactions, the solid product was filtered and thoroughly washed with methanol, followed by treatment with dilute HCl solution, ethanol–water mixture, and ethanol in series to remove unreacted reactants. The purified product was then dried under vacuum at 50 °C for 12 h. The obtained adsorbent was named S_HPEI-QP_ (hyperbranched PEI sponge quaternized and phenyl-functionalized).

### 2.3. Characterizations and Instruments

The obtained materials were characterized by the following analytical techniques. A JSM-7800F scanning electron microscope (Peabody, MA, USA) with SEM-EDS was used to observe the morphology of the S_HPEI-QP_. Thermogravimetric analysis was carried out via a NETZSCH STA 449 F3 thermal analyzer to test the thermal stability in the atmosphere of nitrogen at a heating rate of 10 °C/min in a temperature range of 40–780 °C, with an initial sample weight of approximately 10 mg. Nitrogen adsorption–desorption isotherms were measured at 77 K employing a Quantachrome Autosorb-iQ2 instrument (Quantachrome, Boynton Beach, FL, USA). Fourier transform infrared (FT-IR) spectra were recorded on a Nicolet iS5 spectrometer (Thermo Scientific, Waltham, MA, USA) with a scanning range of 4000–400 cm^−1^ using the KBr pellet method. X-ray photoelectron spectroscopy (XPS) spectra were measured on a Thermofisher ESCALAB 250Xi with Al Kα radiation and were calibrated with the C1s level (284.6 eV) as an internal standard reference. The separation and quantification of IBU were conducted using an Agilent 1200 high-performance liquid chromatography (HPLC) system, which was equipped with a quaternary pump, an online degasser, an autosampler, and a diode array detector (DAD). The mobile phase consisted of Solvent A (methanol) and Solvent B (20 mM ammonium acetate). Chromatographic separation was performed using a gradient elution starting from 30% to 75% Solvent A over 20 min, followed by a rapid return to the initial conditions within 0.5 min, which was then maintained for an additional 3.5 min. The injection volume for all samples was 20 µL. The column temperature was maintained at 25 °C, and detection was conducted at a wavelength of 288 nm.

### 2.4. Batch Sorption Experiments

Working solutions of IBU (50 mg L^−1^) were made by dissolving proper amounts of analyte in MeOH solution (*v*:*v*, 20/80). The sample solutions of the desired concentrations were prepared by further dilutions of working solutions with MeOH solution (*v*:*v*, 20/80) for IBU, and these solutions were freshly prepared each week and stored at 4 °C. Batch adsorption of individual pharmaceuticals was conducted using 8 mg of polymer particles in 100 mL (or 120 mL) of IBU solutions. The mixtures were shaken at 120 rpm for 2 h at 25 °C. After binding, the solutions were filtered through a 0.45 µm PTFE membrane filter.

Adsorption kinetics were obtained by taking the samples after different intervals (0.5, 1, 2, 5, 10, 30, 40, 50, 60, 120, and 240 min) with a fixed initial pharmaceutical concentration (50 mg L^−1^). Adsorption isotherms were produced by varying the initial concentrations from 5 to 100 mg L^−1^, and the pH-effect experiments were carried out at a fixed pharmaceutical concentration (50 mg L^−1^) with varying pHs from 2 to 9. The solution pH was adjusted using 0.2 M NaOH or HCl to the desired value. The ionic strength of the solutions was adjusted by dissolving different amounts of NaCl. Except for the experiments for estimating the pH effect, the solution pH was kept at ~7.

The removal efficiency (*R_e_*%) and adsorption capacity at equilibrium (*q_e_*) were calculated according to Equations (1) and (2), respectively.

(1) $$R_{e}\%=\frac{C_{0}-C_{e}}{C_{0}}\times 100\%$$

(2) $$q_{e}=\frac{C_{0}-C_{e}}{m}\times V$$

where *C*_0_ and *C*_e_ are the initial and equilibrium concentrations of analytes s in solution (mg L^−1^), respectively; *V* is the volume of solution (L); and *m* (g) is the mass of adsorbent.

### 2.5. Regeneration and Recycling Studies

The employed sorbent underwent regeneration at room temperature by agitation in an aqueous–methanol solution (50:50, *v*/*v*) containing 5% NaCl for a period of two hours. Subsequently, the sorbent was subjected to filtration, washed with ethanol, and dried in a vacuum oven to facilitate its reuse. This regeneration protocol was repeated for eight successive cycles.

## 3. Results and Discussion

### 3.1. Characterizations of S_HPEI-QP_

**Figure 2:**
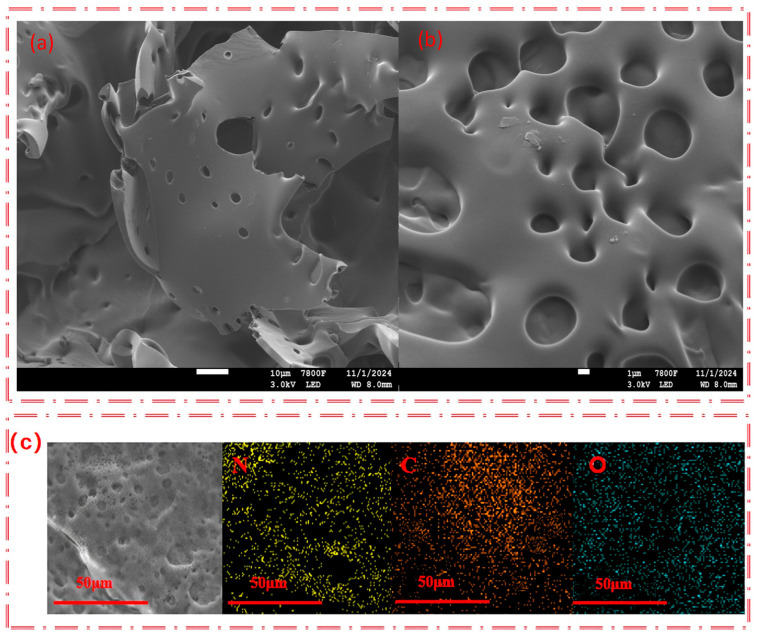
(**a**,**b**) SEM images of S_HPEI-QP_ and (**c**) SEM/EDS mapping showing the distribution of nitrogen (N), carbon (C), and oxygen (O).

**Figure 3:**
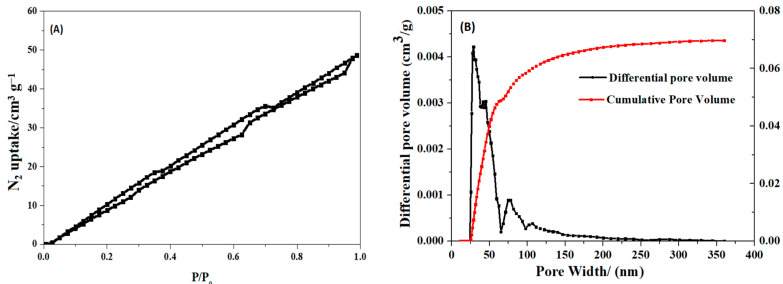
(**A**) N_2_ adsorption–desorption isotherms and (**B**) pore size distribution of S_HPEI-QP_ microspheres.

As shown in Table 2, S_HPEI-QP_ presents a porous structure with pore diameters predominantly ranging from 25 to 100 nm. This porous morphology can increase the specific surface area of the material, provide more adsorption sites, and be more conducive to the adsorption of IBU. From the scanning electron microscope images of the internal morphology (Figure 2a,b), it can be clearly seen that S_HPEI-QP_ well maintains a highly porous and interconnected structure after the bifunctional modification on the sponge. The corresponding energy dispersive X-ray spectrometry (EDS) elemental mapping of N, C, and O (Figure 2c) illustrates a homogeneous distribution of N, C, and O atoms in the skeleton of the dried S_HPEI-QP_. Figure 3 and Table 2 indicate that the BET specific surface area and pore volume of the S_HPEI-QP_ are calculated as 77.2 m^2^ g^−1^ and 0.05 cm^3^ g^−1^ with its pore size distribution ranging from 25 to 100 nm (analyzed by the DFT method).

**Figure 4:**
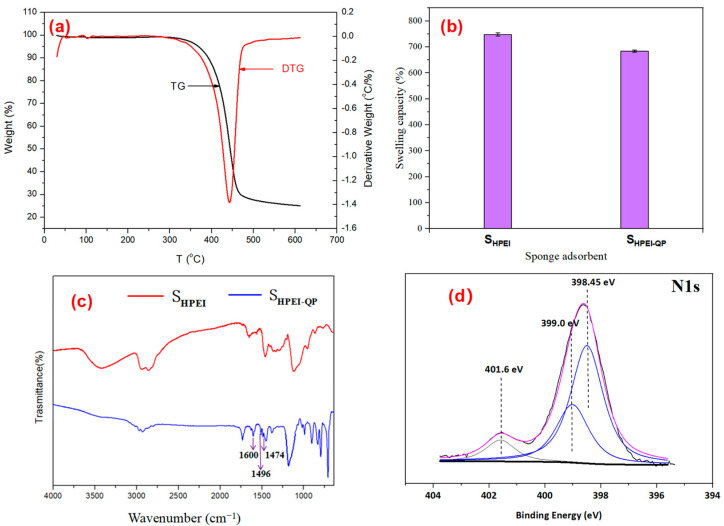
(**a**) TGA curve of S_HPEI-QP,_ (**b**) swelling ratios of S_HPEI_ and S_HPEI-QP_, (**c**) S_HPEI_ and S_HPEI-QP_ FTIR spectra, and (**d**) XPS spectra N1s of S_HPEI-QP_.

Figure 4a shows the thermal degradation curve of S_HPEI-QP_ in a nitrogen atmosphere. A minor weight loss event is evident at approximately 100 °C, characterized by a small, distinct peak in the DTG profile. This negligible mass loss is primarily ascribed to the desorption of absorbed water molecules, consistent with the presence of hydrophilic functional groups on the material surface. The major weight loss peak occurs around 350 °C, which is likely associated with the decomposition and collapse of the S_HPEI-QP_ framework, indicating the excellent thermal stability of S_HPEI-QP_ and a wide range of operating temperatures in water bodies.

The dried sponges were immersed in an excess volume of water at 25 °C for a duration of 24 h to attain equilibrium swelling. Subsequently, the swollen sponges were extracted from the swelling medium, and surface water was carefully removed using filter paper prior to weighing. The swelling ratio (*SR*) was determined according to Equation (3):

(3) $$SR=\frac{\left(W_{s}-W_{d}\right)}{W_{d}}\times 100\%$$

*W_s_* represents the weights of equilibrium swollen;

*W_d_* represents the weights of dry states.

As illustrated in Figure 4b, the swelling ratios of S_HPEI_ and S_HPEI-QP_ reach approximately 748% and 684%, respectively. The high swelling capacity is attributed to their porous three-dimensional structures and hydrophilic frameworks. Notably, the swelling ratio of S_HPEI-QP_ is slightly lower than that of S_HPEI_, and this slight reduction primarily results from the incorporation of functionalized groups in the framework. Excellent swelling capacity of S_HPEI-QP_ facilitates the transport of contaminants within the sponge matrix, thereby fully exposing adsorption sites to target molecules. Consequently, the substantial swelling ability of S_HPEI-QP_ is advantageous for its adsorption performance toward IBU.

To verify the successful simultaneous grafting of two functional groups on S_HPEI_, the FT-IR spectra of S_HPEI_ and S_HPEI-QP_ are presented in Figure 4c. The integration of ether-linked phenyl groups in S_HPEI-QP_ is evidenced by the prominent absorption bands observed at 1600 and 1496 cm^−1^, corresponding to the C=C stretching vibrations of the aromatic ring (R-C_6_H_5_). With comparison of S_HPEI_, the increased intensity of the aforementioned bands in S_HPEI-QP_ indicates the successful graft of RDGE, due to the existence of a benzene ring in RDGE. Furthermore, a newly appearing band at about 1475 cm^−1^ in the spectra of S_HPEI-QP_ is mainly ascribed to the methyl groups in the grafted membranes owing to the addition of trimethylamine with methyl [31]. In Figure 4d, the peak at relatively high binding energy for N1s 401.6 eV corresponds to quaternary ammonium groups, -N^+^(CH_3_)_3_, indicating the successful modification of quaternary amino groups on S_HPEI_ [32].

**Figure 5:**
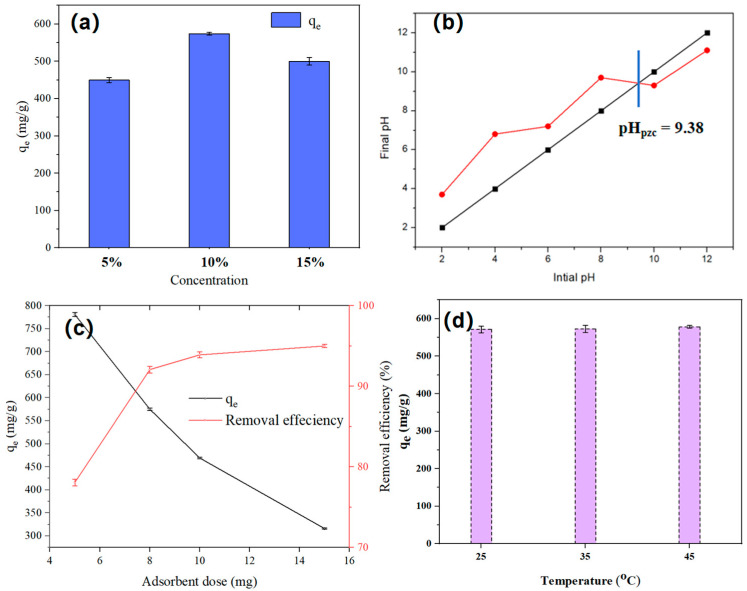
(**a**) PEI concentration of S_HPEI-QP_ (concentration = 50 mg L^−1^, dosage = 0.08 g/L, V = 100 mL, pH 7), (**b**) pH_pzc_ of S_HPEI-QP_ (5 mg S_HPEI-QP_ in 100 mL 0.1 M KCl), (**c**) effect of adsorbent dosage (C_0_ = 50 mg/L, V = 100 mL, pH 7), and (**d**) effect of temperature (concentration = 50 mg L^−1^, dosage = 0.08 g/L, V = 100 mL, pH 7).

As illustrated in Figure 5a, to investigate the effect of grafted PEI concentration on adsorption capacity, three different PEI concentrations (5%, 10%, and 15%) were selected for evaluation. The results indicated that the highest adsorption capacity was achieved at a PEI concentration of 10%. The adsorption capacity at 10% PEI was greater than that at 5%, which can be attributed to the increased amount of PEI grafted onto S_PEI_ at higher concentrations. However, the decrease in adsorption capacity observed when increasing the concentration from 10% to 15% is due to excessive PEI modification, which causes pore blockage and renders internal adsorption sites ineffective, thereby reducing the overall adsorption capacity. Consequently, a PEI grafting concentration of 10% was chosen for all following experiments.

The effect of adsorbent dosage on the adsorption process of IBU onto S_HPEI-QP_ is illustrated in Figure 5c. At the minimum dosage of 5 mg, the adsorption capacity reached its highest value, indicating that the available adsorption sites were fully occupied; however, the removal efficiency was low, suggesting that a considerable amount of the contaminant remained in the aqueous phase. When the dosage increased from 5 mg to 8 mg, although the adsorption capacity decreased, the removal efficiency significantly improved to 92.06%. Beyond a dosage of 8 mg, the adsorption capacity continued to decline, while the removal rate exhibited a slight increase. The reduction in adsorption capacity at dosages exceeding 8 mg is attributed to an excess of adsorption sites relative to the insufficient amount of the contaminant. Therefore, an adsorbent dosage of 8 mg was selected for subsequent experiments, as it optimally balances high adsorption capacity and effective removal efficiency. Moreover, in Figure 5d, an increase in temperature from 25 °C up to 45 °C shows an insignificant improvement in IBU adsorption capacity [33].

### 3.2. Adsorption Properties of S_HPEI-QP_ for IBU

#### 3.2.1. Effect of Solution pH

**Figure 6:**
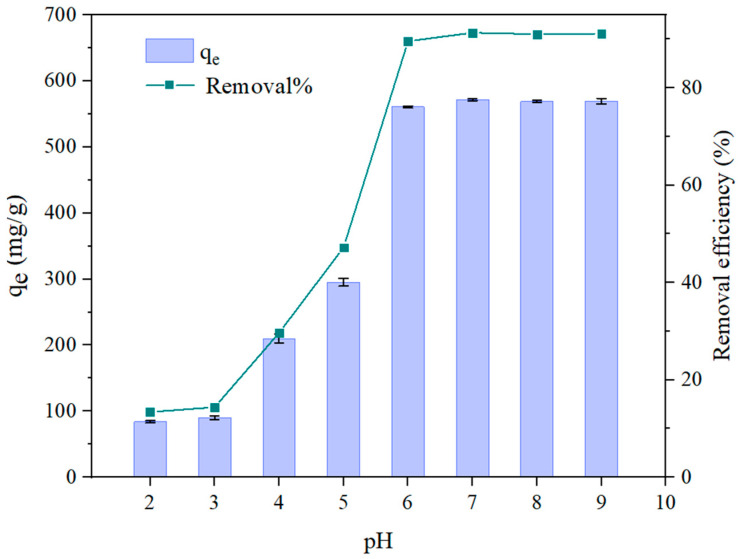
Effect of pH on the adsorption capacity of S_HPEI-QP_ adsorbents (conditions: C_0_ = 50 mg/L, dosage = 0.08 g/L, V = 100 mL, t = 2 h).

In adsorption processes, pH represents a pivotal factor that significantly influences the efficiency of the reaction. Consequently, it is essential to investigate the impact of pH on the removal efficiency of IBU from aqueous solutions. Given that the pKa of IBU is 4.91, the electrostatic interactions between IBU and the adsorbent S_HPEI-QP_ vary with the solution’s pH, thereby affecting the adsorption capacity. As depicted in Figure 6, the adsorption efficiency of IBU markedly increased from 13.4% to 89.6% within the pH range of 2 to 6. The pH of the point of zero charge (pH_pzc_) was found to be 9.38 (Figure 5b), which suggests that the S_HPEI-QP_ has a positive surface charge below pH 9.38 and, thus, strong electrostatic interaction with anionic substances. As shown in Figure 6, within the pH range of 6–9 (>pKa+1~2), IBU is more in its basic form and can be strongly retained by strong anion exchange interaction, especially at the pH range from 7.0 to 9.0, where IBU is fully in its anionic form, while at low pH values (pH = 2, 3, 4, 5), the IBU exists as a protonated and partially ionized form, which weakens the anion exchange interaction. Considering a negligible hydrophobic interaction in the presence of MeOH, the adsorption of IBU was dominated by an electrostatic interaction.

#### 3.2.2. Adsorption Kinetic Study

The most widely used models for adsorption kinetics are the pseudo-first-order kinetic model (4) and the pseudo-second-order kinetic model (5).

(4) $$\ln(q_{e}-q_{t})=\ln q_{e}-k_{1}$$

(5) $$\frac{t}{q_{t}}=\frac{1}{k_{2}q_{e}^{2}}+\frac{t}{q_{e}}$$

In this study, *q_e_* (mg/g) and *q_t_* (mg/g) represent the adsorption capacities at equilibrium and at a given time *t* (min), respectively. The rate constants *k_1_* (1/min) and *k_2_* (g/(mg·min)) correspond to the pseudo-first-order and pseudo-second-order kinetic models, respectively. These parameters are determined from the slope and intercept of linearized plots: ln(*q_e_* − *q_t_*) against *t* for the pseudo-first-order model and *t*/*q_t_* against *t* for the pseudo-second-order model.

**Figure 7:**
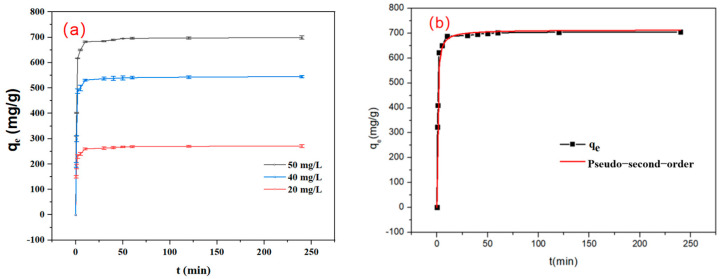
(**a**) Kinetic curves of the adsorption process (conditions: C_0_ = 20 mg/L, 40 mg/L, 50 mg/L, dosage = 0.08 g/L, V = 120 mL, pH 7) and (**b**) fitting for pseudo-second-order equation (conditions: C_0_ = 50 mg/L, dosage = 0.08 g/L, V = 120 mL, pH 7).

As shown in Figure 7a, the adsorption of IBU at different concentrations (20, 40, 50 mg_IBU_/L) reached equilibrium within an extremely short period of time (<20 min), which may be ascribed to the abundant pore structure of the adsorption material. This adsorption material with uniform and large pores enables IBU molecules to rapidly diffuse into the pores and be adsorbed on the surface within a short period. Within the initial 20 min, the adsorption process is rapid, attributable to the abundance of available active binding sites on the adsorbent surface. Between 20 and 120 min, the adsorption rate gradually approaches equilibrium, suggesting that the majority of the adsorption sites have become occupied, although a limited number of vacant sites remain capable of adsorbing IBU. Equilibrium in the adsorption of IBU is attained after 20 min. The adsorption kinetics data were analyzed using pseudo-first-order and pseudo-second-order kinetic models (see Table 3). As shown in Figure 7b, the adsorption data of IBU are in perfect agreement with the pseudo-second-order model, as evidenced by the high correlation coefficient (R^2^ = 0.997), indicating that the adsorption of IBU on S_HPEI-QP_ is predominantly governed by a chemical adsorption process [34].

#### 3.2.3. Adsorption Isotherm Study

To further elucidate the adsorption characteristics and evaluate the maximum adsorption capacity, the adsorption behavior of IBU on S_HPEI-QP_ was investigated using the Langmuir and the Freundlich isotherm models. The well-established Langmuir (Equation (6)) and Freundlich (Equation (7)) nonlinear models were employed to simulate the adsorption data, which were based on the assumptions of monolayer adsorption on the homogeneous surface and multilayer adsorption on the heterogeneous surface, respectively. The Langmuir model assumes monolayer adsorption on a homogeneous surface without interactions between adsorbed molecules, thereby being appropriate for single-layer adsorption on uniform surfaces. In contrast, the Freundlich model is based on multilayer adsorption on heterogeneous surfaces, applicable to adsorption on non-uniform surfaces.

(6) $$q=q_{m}\frac{K_{L}C_{e}}{1+K_{L}C_{e}}$$

(7) $$q=K_{f}C_{e}^{1/n}$$

where *q_m_* (mg g^−1^) is the maximum adsorption capacity, *K_L_* and *K_f_* (L mg^−1^) are the Langmuir and Freundlich affinity coefficients, respectively, and *n* (unitless) represents the energetic heterogeneity factor related to adsorption intensity.

**Figure 8:**
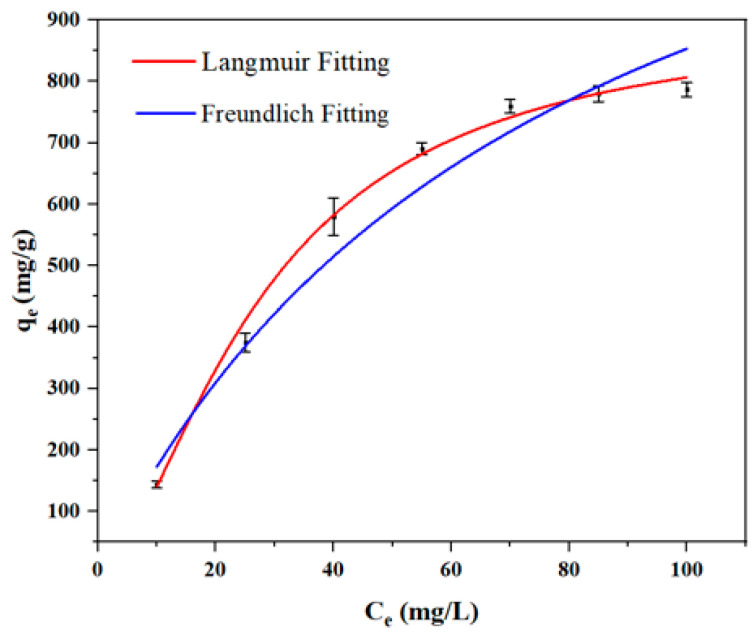
Fitting for the Langmuir equation and the Freundlich equation (conditions: dosage = 0.08 g/L, V = 120 mL, pH 7, t = 2 h).

To gain a deeper insight into the adsorption properties and to quantify the maximum adsorption capacity, the adsorption behavior of IBU on S_HPEI-QP_ was evaluated using the Langmuir and the Freundlich isotherm models. The Langmuir model assumes monolayer adsorption occurring on a homogeneous surface without interactions between adsorbed molecules, whereas the Freundlich model describes multilayer adsorption on heterogeneous surfaces. As illustrated in Figure 8 and Table 4, the adsorption isotherm data for IBU on S_HPEI-QP_ exhibit a superior fit to the Langmuir model, as evidenced by a higher correlation coefficient compared to the Freundlich model. This finding suggests that chemisorption involving monolayer adsorption on a uniform surface is the dominant mechanism in this system. From the isotherm analysis, the maximum adsorption capacity (q_m_) of IBU was calculated to be 905.73 mg/g, which surpasses the capacities reported for other adsorbents in previous studies (refer to Table 5). These results highlight the promising potential of S_HPEI-QP_ for the efficient removal of IBU from neutral and mildly acidic aqueous media.

### 3.3. Regeneration and Reusability

**Figure 9:**
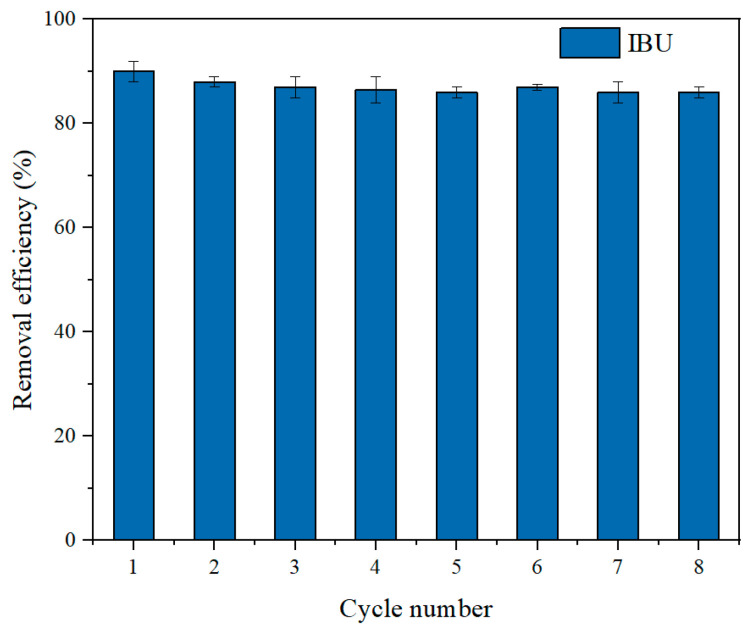
IBU removal efficiencies (%) in eight subsequent adsorption–desorption cycles (conditions: C_0_ = 50 mg/L, dosage = 0.08 g/L, pH = 7, V = 120 mL, t = 120 min).

Reusability is a critical criterion for assessing the economic feasibility of adsorbent materials. Typically, strong anion exchange adsorbents are regenerated using concentrated sodium hydroxide and sodium chloride solutions. Due to the hydrophobic nature of IBU, nonpolar organic solvents such as methanol were commonly employed to disrupt hydrophobic interactions between IBU and hydrophobic ligands. In this study, the adsorbent was eluted using a 4% NaOH aqueous/methanol solution (50/50, *v*/*v*) under agitation on a shaker for 2 h. After eight adsorption–desorption cycles, the regenerated adsorbent maintained over 80% of its initial adsorption efficiency capacity, as illustrated in Figure 9. The gradual decline in adsorption capacity with repeated cycles is primarily attributed to incomplete desorption of target molecules occupying binding sites, as well as adsorbent loss during the elution process. The excellent regeneration performance of S_HPEI-QP_ further substantiates its potential as an environmentally friendly adsorbent for the effective removal of non-steroidal anti-inflammatory drugs from aqueous solutions in practical application prospects. The S_HPEI-QP_ developed in this study demonstrates outstanding adsorption capacity compared to the reported adsorbents in Table 5. Additionally, its excellent regeneration performance, superior to the reported adsorbents [35,36,37], can reduce the required amount of adsorbent. The adsorption process occurs under mild conditions (ambient temperature and neutral pH), eliminating the need for complex equipment and thereby decreasing energy consumption and operational costs during application.

**Table 5:** IBU adsorption performance of the S_HPEI-QP_ and other reported adsorbents.

Adsorbents	Optimum pH	Dosage (g L^−1^)	S_BET_ (m^2^ g^−1^)	Q_max_ (mg g^−1^)	Ref.
S_HPEI-QP_	5–9	0.08	77.2	905.73 for IBU	This study
Carbon nanospheres (CNS)	6	0.8	359	356.89 for IBU	[35]
Magnetic anion exchange resin	6–8	1.0	3.62	47.4 for IBU	[38]
UiO-66(Zr)	3	0.25	1139.2	729.92 for IBU	[39]
SBA-15	3	10	879	0.41 for IBU	[40]
Mesoporous silver impregnated granules of aluminum mineral	5–7	1.33	268	8.24 for IBU	[41]
Iron-incorporated pomegranate husk carbon (NPH)	8	0.05	190	39.77 for IBU	[42]
Magnetic carboxylic multiwalled carbon nanotube	1–10	0.0125	2.38	370.52 for IBU	[43]

## 4. Conclusions

A novel adsorbent material featuring porous S_HPEI-QP_ was synthesized via a low-temperature polymerization method, exhibiting rapid mass transfer capabilities and the advantages of densely functionalized dendritic molecules. This material demonstrates not only a remarkable adsorption capacity (with an adsorption amount of 905.73 mg/g for IBU) but also a rapid adsorption rate, achieving equilibrium within 20 min. The adsorbent integrates the hydrophobicity of phenyl groups with the strong anion exchange functionality of quaternary ammonium groups, thereby conferring significant selectivity and adsorption efficiency toward the polar anion IBU in aqueous solutions. As a high-capacity and highly selective adsorbent, this material fundamentally addresses the key limitations of conventional adsorbents, which typically exhibit low efficiency and poor selectivity for polar ionic compounds. Furthermore, the primary adsorption mechanism for IBU was investigated to be the strong electrostatic interactions between quaternary ammonium groups in S_HPEI-QP_ and deprotonated amines in IBU. Importantly, the three-dimensional skeletal structure facilitates mass transfer, resulting in ultrafast adsorption kinetics. After eight adsorption–desorption cycles, the adsorption efficiency decreased by only 4%, indicating that S_HPEI-QP_ is a reusable adsorbent and is suitable for the removal of IBU from water. Overall, S_HPEI-QP_ represents a sustainable, efficient, reusable, cost-effective, and eco-friendly adsorbent, capable of effectively and selectively removing potentially toxic anionic pollutants from complex aqueous environments. The strength of electrostatic interactions plays a critical role in governing the dominant adsorption process.

## Figures and Tables

**Table 1:** IBU chemical, physical, and structural properties.

Pharmaceutical	pKa	CAS Number	Log P	Water Solubility (mg L^−1^)	Structure
IBU	4.91	15687-27-1	3.97	21	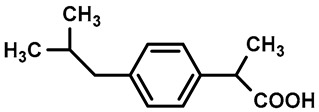

**Table 2:** Textural properties for the sorbents.

	S_BET_ (m^2^ g^−1^)	Pore Volume (cm^3^ g^−1^)	Pore Size (nm)
S_HPEI-QP_	77.2	0.05	25–100

**Table 3:** Adsorption kinetic parameters of S_HPEI-QP_ for IBU removal.

		Model					
		**Pseudo-First-Order**			**Pseudo-Second-Order**		
Material	q_e,exp_ (mg/g)	q_e_ (mg/g)	K_1_ (min^−1^)	R^2^	q_e_ (mg/g)	K_2_ (g/mg·min)	R^2^
S_HPEI-QP_	705.11	592.54	0.08	0.889	713.44	0.0024	0.997

**Table 4:** Characteristic parameters of the Langmuir and the Freundlich isotherms for the adsorption of IBU onto S_HPEI-QP_.

		Model						
		**Langmuir Isotherm**				**Freundlich Isotherm**		
Material	q_e,exp_ (mg/g)	q_m_ (mg/g)	K_L_ (L mg^−1^)	R_L_	R^2^	K_f_ (L mg^−1^)	n	R^2^
S_HPEI-QP_	786.66	905.73	0.156	0.0791	0.991	53.1	1.43	0.977

## Data Availability

The original contributions presented in this study are included in the article. Further inquiries can be directed to the corresponding author.
